# 4034 Can Connections IN Health become a research-based model to improve health outcomes through community health coalitions?

**DOI:** 10.1017/cts.2020.231

**Published:** 2020-07-29

**Authors:** Lily Darbishire, Sarah Wiehe, Dennis Savaiano

**Affiliations:** 1 Purdue University; 2 Indiana University School of Medicine

## Abstract

OBJECTIVES/GOALS: Connections IN Health’s goal is to coordinate, integrate, and enrich health coalition work through extended connections among community and academic stakeholders within and across coalitions and geographies within Indiana. We aim to evaluate stakeholder connections to assess coalition effectiveness and the quality of partnership networks. METHODS/STUDY POPULATION: We will collect data longitudinally to evaluate Connections IN Health using a unique triangulation of effectiveness surveys, social network analysis, and health data. Cross-sectional functioning and social network analysis surveys were distributed to coalition members before the transition to Connections IN Health engagement (baseline) and will be distributed again each year thereafter to identify changes in coalition perceived effectiveness and changes in the structure/nature of partnership networks after implementation of the partnership. We plan to utilize publicly available health data to measure proximal changes in health outcomes at the neighborhood level and use Pearson’s correlations to check for associations between perceived coalition effectiveness and health outcomes. RESULTS/ANTICIPATED RESULTS: We found low baseline scores in perceived effectiveness, especially in the areas of leadership, operational understanding, and satisfaction, from the coalition members. From our social network analysis, we found relatively low cohesion scores (measured as network density) among each of the coalition networks, and even lower scores for collaboration among coalition members. We expect to see positive increases in perceived coalition effectiveness, as well as an increase in the density and level of collaboration among coalition networks as Connections IN Health develops. Finally, we expect to see positive changes in proximal health outcomes associated with our measures of coalition effectiveness. DISCUSSION/SIGNIFICANCE OF IMPACT: The results of our project will be distributed back to the coalition leaders and members in order to sustain and improve the coalitions. The visualization of the coalition member’s network can be used to demonstrate opportunities for enhanced partnerships and collaboration.

